# The Influence of Road Geometry on Vehicle Rollover and Skidding

**DOI:** 10.3390/ijerph17051648

**Published:** 2020-03-03

**Authors:** Yanna Yin, Huiying Wen, Lu Sun, Wei Hou

**Affiliations:** 1 School of Civil Engineering and Transportation, South China University of Technology, Guangzhou 510641, China; yyn1241085280@163.com (Y.Y.); hywen@scut.edu.cn (H.W.); 2 Department of Civil and Environmental, University of Maryland, College Park, MD 20742, USA; 3 Qilu Transportation Development Group; Jinan 250000, China; blxmb2015@163.com

**Keywords:** The most unfavorable road models, Rollover, Skidding, The critical wheel

## Abstract

This paper analyzes the influence of single and combined unfavorable road geometry on rollover and skidding risks of D-class mid-sized sport utility vehicles (SUVs) with front-wheel drive for roads with design speeds at 80 km/h. A closed-loop simulation model of human-vehicle-road interactions is established to examine the systematic influence of road geometry on vehicle rollover and skidding. The effects of different road geometry on rollover and skidding on SUVs are studied for pavement surface with good and poor friction when vehicles are in the action of steady state cornering. The rollover and skidding risks of the most unfavorable road segments are assessed. The critical wheel is defined by the threshold of skidding during curve negotiation. The results found that SUVs are not easy to rollover on the most unfavorable roads, regardless of good or poor friction of pavement surface. The safety margin of rollover is greater than that of skidding. The safety margin of skidding is minimal on poor friction roads. Therefore, for the sake of driving safety, it is not recommended to design the roads with these unfavorable road geometry combinations

## 1. Introduction

Highway safety is an issue of great concern to transportation professionals all over the world [1,2,3,4,5,6,7,8,9,10,11,12]. Despite the decline in highway fatalities between 20012014, the National Highway Traffic Safety Administration’s (NHTSA) Fatality Analysis Reporting System (FARS) reported that fatal accidents increased between 2015–2016 [13]. In fact, more than 25 percent of fatal crashes are associated with horizontal curvature [14]. Roads cause great safety risk due to the difference in road alignment, which pose the risk that drivers deviate from the original road. If the driver fails to adjust the speed and driving direction in time, the vehicle will roll-over and skid, which has high potential for a severe crash.

According to the statistics of National Highway Traffic Safety Administration [13], more rollovers accidents were caused by single-vehicle crashes than that of multiple-vehicle crashes in 2016 in the United States. About three-quarters of curve-related fatal crashes involve single vehicles leaving the roadway and striking fixed objects (trees, utility poles, rocks, etc.) or rolling over [14]. Vehicle rollover is a typical fatal accident at a bend [15,16].

Vehicle’ rollover can generally be divided into two categories [17]. One is the maneuver- induced rollover; the other is the tripped rollover. The former refers to the rollover caused by the vehicle’s lateral acceleration exceeding a certain limit, which makes the vertical counter-force of the wheels on the inside of the vehicle zero. The latter is that the vehicle initiates skidding and strikes obstacles on the road tripping the vehicle over obstacles. Many factors may cause vehicle rollover; this includes vehicle structure, driver conditions, and road conditions [17]. In this paper, two kinds of rollovers will be presented: non-tripped rollovers that are maneuver-induced and tripped rollovers caused by skidding. It is difficult to determine whether vehicles will be tripped over or not after the occurrence of skidding, but the vehicles will be in lateral instability and prone to collision and rollover.

The above analysis shows that rollover and skidding are typical traffic safety problems. However, the shortcomings of existing studies are as described below.

The research methods of the influence of road factors on vehicle rollover are mostly concentrated on accident-prone incidents [16,18,19,20,21,22,23,24]. It is difficult to determine the underlying causes behind the formation of black spots, and the results are not intuitive. In addition, this method cannot exclude the influence of bad weather (ice, snow, rain, and fog) on the driver, indirectly affecting the possible rollover of the vehicle. There are relevant references [15,25,26,27,28,29,30,31,32] to study the influence of road geometry on the dynamic indicators of vehicle rollover and skidding. However, no researchers have ever studied the impact of unfavorable geometric combinations on vehicle’ rollover and skidding. These types of roads are the most important problem in accidents. In the existing specifications [33], the minimum turning radius calculated by the point mass model neglects influence of vehicle type, centroid position, grade of road, etc. The point mass model does not consider the effect of rollover.

Traffic accidents are usually affected by the comprehensive human-vehicle-road interaction factor. To this end, the human-vehicle-road simulation model is developed by the vehicle simulation software-CarSim(Mechanical Simulation Corporation, Michigan, America). This paper takes the SUV as the research object due to its high center of gravity and increasing popularity, which is more prone to rollover than passenger cars [34,35]. This research focuses on systematically studying the influence of unfavorable roads on vehicle rollover and skidding. The effects of different road geometric combinations on rollover and the rollovers of SUVs are analyzed. The safety of road design parameters is evaluated.

The contributions of this paper are three-fold. Firstly, a new method is proposed to define the most critical unstable wheel. Secondly, combinations of unfavorable road geometries are defined and proposed according to Chinese technical specification of highway geometric design. Thirdly, a closed-loop human-vehicle-road interaction model is established to assess the risks of rollover and skidding of SUVs.

## 2. Human-Vehicle-Road Closed Loop Simulation Model

### 2.1. Road Model

**Figure 1:**
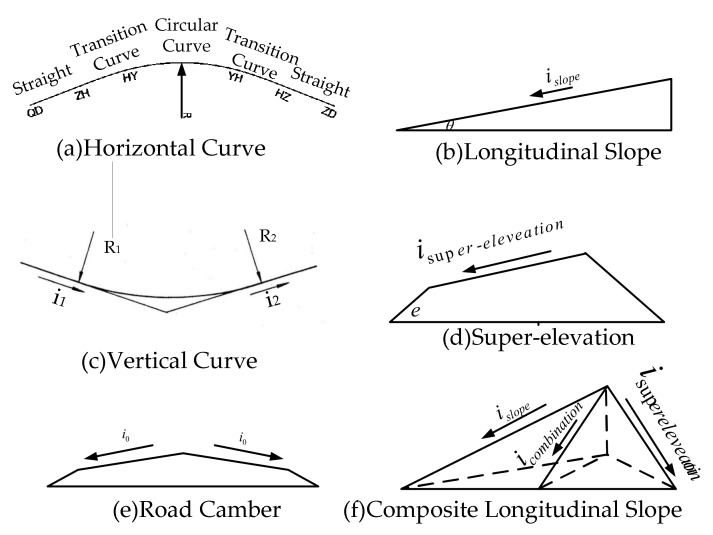
Road geometric parameters. (**a**) horizontal curve; (**b**) longitudinal slope; (**c**) vertical curve; (**d**) super-elevation; (**e**) road camber; (**f**) composite longitudinal slope.

Based on our previous research [25,26] and technical specification for geometric road design of China, road segments of single unfavorable geometry and road segments of multiple unfavorable road geometry combinations are established. The geometric road alignment is used as an input module, including the horizontal alignment, the longitudinal alignment, the cross slope of them, and their combination. The horizontal curve shown through Figure 1a is usually connected by straight lines, transition curves, and circular curves, where R is the radius of the circular curve. The upslope and downslope along the direction of the road is called profile grade [36], which includes straight and vertical curves (shown through Figure 1b,c). $i_{slope}$ denotes the grade of the longitudinal slope. In geometric road design, super-elevation, part of the cross slope shown in Figure 1d, is usually designed to offset part of the centrifugal force used when a vehicle is traversing a curve. The super-elevation is denoted as $i_{\sup erelevation}$. In order to facilitate pavement drainage, the road is designed into an arch with a central inclination to both sides, which is called the road camber [37] (shown in Figure 1e). The inclination is denoted as $i_{0}$. When the super-elevation overlaps with the longitudinal slope, it can be called the composite longitudinal slope of the road (shown in Figure 1f). The calculation formula is as follows [25,26]:

(1) $$i_{combination}=\sqrt{i_{\sup erelevation}^{2}+i_{slope}^{2}}$$

Because the longitudinal slope and super-elevation of road is very small, the angle of cross slope of road is approximately equal to its super-elevation, $e\approx i_{\sup erelevation}$. For the same reason, the angle of longitudinal slope is approximately equal to the grade, $\theta \approx i_{slope}$, as well as the composite longitudinal slope angle $\theta_{c}\approx i_{combination}$ [25,26].

### 2.2. Vehicle Model

#### 2.2.1. Full Vehicle Model

With the development of vehicle dynamics research, the complexity of vehicle model is higher and higher, from the initial mass point model and 2 degrees of freedom model to the later 8 degrees of freedom model and 11 degrees of freedom model [25,38,39,40]. Simulation software includes ADAMS from mechanical dynamics Inc. (now incorporated into Mechanical Simulation Corporation, Michigan, America), CarSim, and TruckSim from mechanical simulation Corporation (Michigan, America). The simulation of ADAMS software can be used to predict the mechanical system performance, motion range, collision detection, peak load, and calculate the input load of finite elements. The model is complex, the simulation quality is high, but the simulation speed is slow. CarSim and TruckSim are multi-body dynamic simulation software based on parameters. The simplified model has 27 degrees of freedom, with high reliability and efficiency. TruckSim is mainly based on multi-body dynamic simulations of trucks.

**Figure 2:**
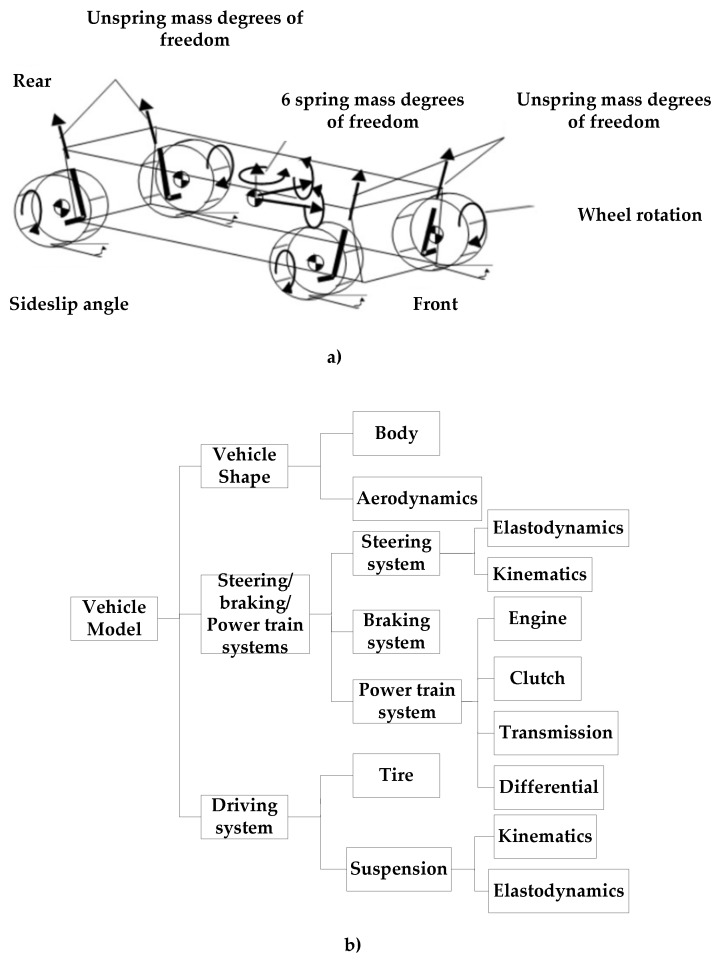
(**a**) 27-Degree of Freedom (DOF) Vehicle Model. (**b**) CarSim Vehicle Structure.

In this paper, CarSim is used to establish the vehicle model. CarSim software abstractly simplifies the vehicle into 10 parts as shown in Figure 2a: one part is the body, four parts are unspring mass, four parts are rotating wheels, and one part is the engine crankshaft. The simplified model includes 27 degrees of freedom: three spring mass moving degrees of freedom (x, y, z), three spring mass rotating degrees of freedom (X, Y, Z), four unspring mass degrees of freedom, four wheel rotating degrees of freedom, one degree of freedom of transmission, eight degrees of freedom of transient characteristics of tires, and four degrees of freedom of braking pressure. Specifically, CarSim’s vehicle model, as shown in the following Figure 2b, includes seven subsystems: car body, aerodynamics, transmission assembly, brake system, steering system, tire, suspension. In this paper, a D-class mid-sized, front wheel drive SUV is selected as the research object. The specific parameters of the vehicle are shown on Table 1 [25].

#### 2.2.2. Tire Model

This paper adopts the “Magic Formula“ tire model with high fitting accuracy [40,41], which is put forward by Professor Pacejka. The general expression of “Magic Formula” is:

(2) $$y=Dsin\left\{Carctan[Bx-E(Bx-arctanBx)]\right\}$$

In the formula, y can be a longitudinal force, a lateral force, or a return moment, while the independent variable can represent the sideslip angle or longitudinal slip rate of the tire in different cases. Similar to Petegem and Farmer [21,22], we only consider normal driving condition where tires with adequate inflation are in good contact with pavement surface, and therefore, the horizontal and vertical zero drift of tires can be ignored. Using the algebraic polynomial method to fit the longitudinal force formula under pure slip condition, we are given:

(3) $$F_{x}(l)=D_{x}sin(C_{x}arctan(B_{x}l-E_{x}(B_{x}l-arctan(B_{x}l))))$$

where

$C_{x}=a_{0};\;D_{x}=(a_{1}F_{z}+a_{2})F_{z}\;B_{x}=(a_{3}F_{z}^{2}+a_{4}F_{z})exp(-a_{5}F_{z})/(C_{x}D_{x})\;E_{x}=a_{6}F_{z}^{2}+a_{7}F_{z}+a_{8}$

The lateral force formula under pure slip condition as follows:

(4) $$F_{y}(a)=D_{y}sin\left\{C_{y}arctan[B_{y}a(1-E_{y})+E_{y}arctan(B_{y}a)]\right\}$$

where

$C_{y}=b_{0}\;D_{y}=(b_{1}F_{z}+b_{2})F_{z}\;B_{y}=b_{3}sin[b_{4}arctan(b_{5}F_{z})]/(C_{y}D_{y})\;E_{y}=b_{6}F_{z}^{2}+b_{7}F_{z}+b_{8}$

where *α* is the wheel sideslip angle, *λ* is the wheel slip rate, *F_x_(λ)* is the longitudinal force calculated under pure slip condition, *F_y_(α)* is the tire sideslip force under pure sideslip movement, and *F_z_* is the tire vertical force. The values of fitting parameters *a*, *b* are shown in Table 2 [42].

### 2.3. Driver Model

In this study, a closed-loop simulation optimal preview model [43,44] for trajectory correction is used. The driver obtains the road and vehicle information through “preview” and “perception”. Based on the information, the driver makes a judgment, adjusts the vehicle’s motion state, to form a closed circulation system. The station for a target location is:

(5) $$S_{targ,i}=S+\frac{iV_{x}T}{m}$$

The horizontal geometry of the road is provided as a sequence of X and Y coordinates that define the road’s centerline. For any given value of S, there is a unique set of X and Y coordinates. Station is defined as a function of X and Y by connecting the points with straight lines. For each pair of X-Y coordinates, a corresponding increment of S is computed by using Pythagorean Theorem. This new increment is added to the previous value of S.

(6) $$S_{i}=S_{i-1}+\sqrt{{\left(X_{i}-X_{i-1}\right)}^{2}+{\left(Y_{i}-Y_{i-1}\right)}^{2}}$$

**Figure 3:**
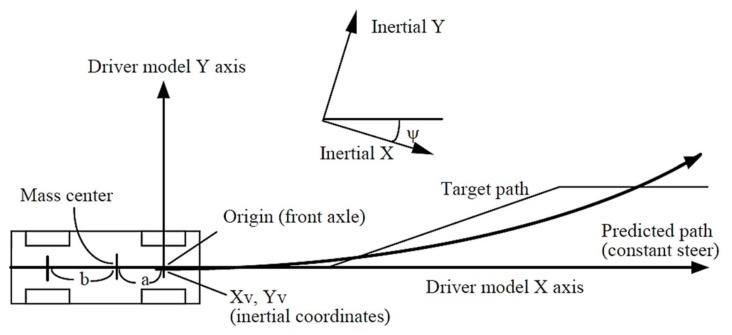
Axis system of steering controller.

The calculation of the controller uses a special coordinate axis system, as shown in Figure 3. In the coordinate axis, the center of the vehicle front axle is *x* = 0, *y* = 0. The yaw of a vehicle is defined as *ψ* = 0. The X and Y axis are aligned with the longitudinal and lateral axes of the vehicle. In the coordinate axis of the driver’s controller, the motion of the vehicle is predicted based on these axes. The axis is fixed in the inertial reference and rotated *ψ* based on the inertial axis.

The target lateral translation in this coordinate system is calculated by first getting the inertia X and Y coordinates of the path as the station function for the target position (S_targ_), and then applying the transformation:

(7) $$Y_{t\arg}=[Y(S_{t\arg})-Y_{V}]cos(\psi )-[X(S_{t\arg})-X_{V}]sin(\psi )$$

Within the coordinate range of the steering controller, the vehicle is always at the origin of the axis, as shown in Figure 3. The time is 0, and the target path is known from time 0 to preview time, T.

## 3. Vehicle Safety Margin of Rollover and Skidding

**Figure 4:**
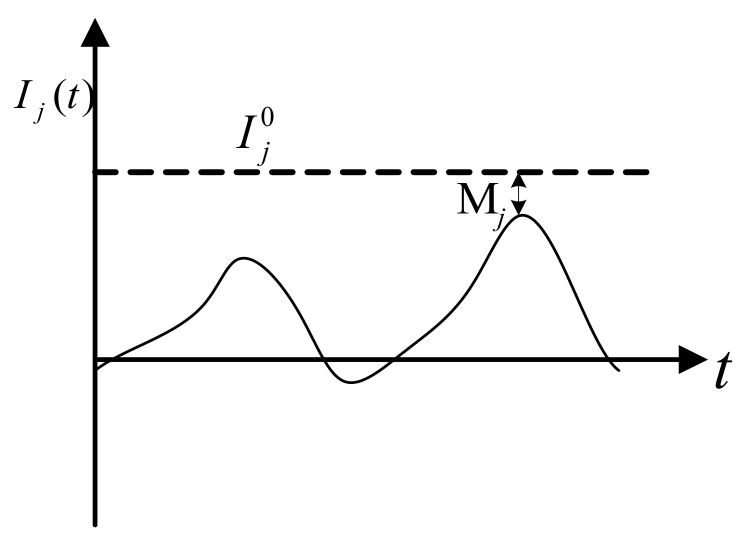
Time history curve of dynamic index j^th.^

The safety margin used in our previous research [26,31] is adopted as a variable to reflect the extent of the index approaching the threshold value. As shown in Figure 4, the curve of the vehicle dynamic index *I_j_(t)* varies with time. It can be seen how the larger vehicle dynamics index *I_j_(t)* functions; the closer it approaches the threshold value, the greater possibility of rollover or skidding becomes. Therefore, the safety margin *M_j_* can be used to measure the risk of vehicle rollover and skidding. The formula is as follows:

(8) $$M_{j}=I_{j}^{0}-max(\left|I_{j}\left(t\right)\right|)0\leq M_{j}\leq I_{j}^{0}$$

### 3.1. Safety Margin of Vehicle Rollover

Lateral acceleration is selected in [26,31] as a risk indicator for vehicle rollover. Based on the definition of rollover [17], through the quasi-static model of rigid vehicles (ignoring the elastic deformation of the suspension and tire of the vehicle), the torque formula of the contact point between the wheel and the ground, on the inside and outside of the roll plane, is as follows:

(9) $$ma_{y}h_{g}-mg\beta h_{g}+F_{Zi}B-\frac{1}{2}mgB=0$$

(10) $$\frac{a_{y}}{g}=\frac{\frac{1}{2}B+\beta h_{g}-\frac{F_{Z}{}_{i}}{mg}B}{h_{g}}=\left[\frac{1}{2}-\frac{F_{Zi}}{mg}\right]\frac{B}{h_{g}}+\beta$$

when *F_Zi_* = 0, the vehicle does not balance in the roll plane, and begins to rollover. The lateral acceleration is called the rollover threshold, when the vehicle starts to rollover. This can be given by the following formula:

(11) $$\frac{a_{y}}{g}=\frac{B}{2h_{g}}+\beta$$

Obviously, when the ramp angle is *β* = 0, the rollover threshold is *a_y_/g = B/*2*h_g_*. The parameters of the SUV are brought into the formula to get the result *a_y_ =* 1.2*g*. The quasi-static model is reasonable only when the lateral acceleration changes slowly. The threshold value of vehicles’ transient rollover is smaller than that of the quasi-static model. For sedans and multi-purpose vehicles, the rollover threshold is about 30% lower. Therefore, the threshold value of rollover here is $a_{y}^{0}=0.84g$, and the corresponding rollover safety margin is:

(12) $$M_{2}=a_{y}^{0}-max(\left|a_{y}^{}(t)\right|)0\leq M_{2}\leq a_{y}^{0}$$

### 3.2. Safety Margin of Vehicle Skidding

According to the definition of skidding [17], the lateral force coefficient of wheels is taken as the indicator of vehicle skidding. The lateral friction coefficient of a vehicle is defined as [26,31]:

(13) $$\mu_{l}=max(\left|\frac{F_{yi}}{F_{Zi}}\right|)\;i=1,2,3,4$$

where *F_yi_* is the tire’s lateral force, *F_Zi_* is the tire’s vertical force and *i* = 1,2,3,4 stands for left front wheel, left rear wheel, right front wheel, and right rear wheel, respectively.

When the lateral force of any wheel is greater than the ground adhesion force, the vehicle will skid [45]. The threshold value is defined as $\mu_{l}^{0}$ when the skidding is about to occur, that is, the maximum lateral friction coefficient (*f_Rmax_*) that the pavement can provide. Thus, the corresponding safety margin is:

(14) $$M_{3}=\mu_{l}^{0}-max(\mu_{l})\;0\leq M_{3}\leq \mu_{l}^{0}$$

According to the literature [46], this is the relationship between the maximum lateral friction coefficient and the peak friction coefficient (*f_Tmax_*) is *f_Rmax_ = 0.925f_Tmax_*. This paper studies good (*f_Tmax_ = 0.85*) and poor (*f_Tmax_ = 0.50*) friction pavement [17]. Therefore, the maximum lateral friction coefficient is approximately equal to 0.78 on good friction pavement, that is, the skidding threshold value is $\mu_{l}^{0}\approx 0.78$. When $\;\mu >\mu_{l}^{0}$, the vehicle will commence to skid. Correspondingly, the skidding threshold value of poor friction pavement is obtained. The above-mentioned friction values refer to the ‘Highway Engineering Technology Standard’ (JTG B 01-2014) of China (shown in Table 3).

The longitudinal friction coefficient of a wheel refers to the ratio of the longitudinal force to the vertical force of each wheel at a certain time, and takes the absolute value to it. The equation is as follows:

(15) $$\mu_{s}=\left|\frac{F_{xi}}{F_{zi}}\right|$$

where *μ_s_* is the longitudinal friction coefficient, *F_xi_* is the longitudinal force of each wheel, and *F_zi_* is the vertical force of each wheel.

## 4. Numerical Analysis

### 4.1. Determination of the Most Critical Wheel

In the Chinese “Highway Engineering Technology Standard” (JTG B 01-2014), road geometry is always designed under a specific design speed, which is determined based on the level of the road (e.g., freeway, level 1, level 2). This study concentrates on a design speed of 80 km/h. Road segments composed of the single unfavorable road geometry are established with a design speed of 80 km/h. The parameters of the road segments are shown in Table 4. The turning direction is left and the grade is upgrade.

Case 1: The road segment is a straight line, without a grade and a super-elevation, and the road camber is 2%. This road segment is a reference for the purpose of comparison.

Case 2: The road segment is a straight line with the grade (maximum allowable grade specified in the Standard for a design speed of 80 km/h), with road camber but without super-elevation.

Case 3: The road segment is a curve, with a super-elevation but without grade. The radius of the circular curve is the minimum limit radius specified in the Standard for a design speed of 80 km/h. The super-elevation is the maximum super-elevation specified in the Standard for a design speed of 80 km/h.

Case 4: The road segment is a curve with a grade (the maximum grade specified in the standard for a design speed of 80 km/h), and a super-elevation. According to the Standard for a design speed of 80 km/h, the composite grade is no more than 10.5%. Thus, the maximum super-elevation can be uniquely obtained. The radius of circular curve is the minimum limit radius specified in the Standard for a design speed of 80 km/h.

**Figure 5:**
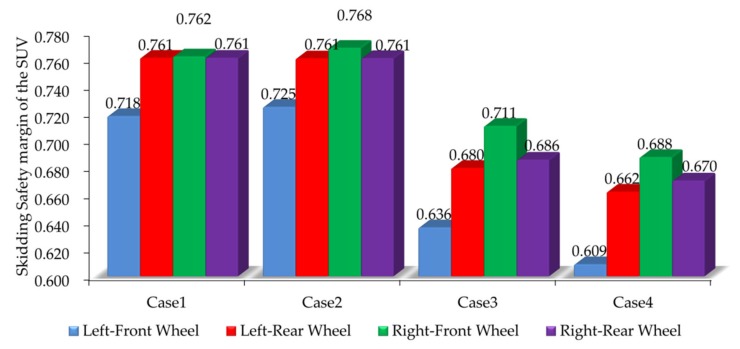
Skidding safety margins of four wheels under different road segments.

The skidding indices of the SUV under four road segments are obtained through CarSim simulation. The skidding safety margins of four wheels are shown in Figure 5.

When comparing the left front wheel of Case 1 with Case 2 in Figure 5, it can be seen that the grade can increase the skidding safety margin, but the increase is very small. The influence of grade on the skidding safety margin is further studied below. Comparing the left-front wheel of Case 1 with Case 3 in Figure 5, it can be seen that although the super-elevation of the SUV can offset part of the centrifugal force of the vehicle moving on the curve, the curve still has a great influence on the safety margin of skidding, which can reduce the safety margin.

Figure 5 shows that the skidding safety margins of four wheels under the same road model are not much different, but the skidding safety margin of the left-front wheel (front-inner wheel) is the smallest under any of the four road models. This means that four wheels do not always skid at the same time even when the threshold value of skidding is reached. This shows that the front-inner wheel is prone to skidding. Gauss [47] considered that the pavement friction reserves were distributed in the longitudinal and transverse directions. During a curve negotiation, some longitudinal friction is occupied by demanded lateral friction, which is produced by centrifugal force. The following equation applies; the upper limit is called the impending skid conditions:

(16) $$N={\left(\frac{f_{T}}{f_{T,max}}\right)}^{2}+{\left(\frac{f_{R}}{f_{R,max}}\right)}^{2}\leq 1$$

where *f_T_* is the longitudinal friction demand, *f_T_*_,*max*_ is the maximum longitudinal friction, *f_R_* is the side friction factor demand, and *f_R,max_* is the maximum side friction.

**Figure 6:**
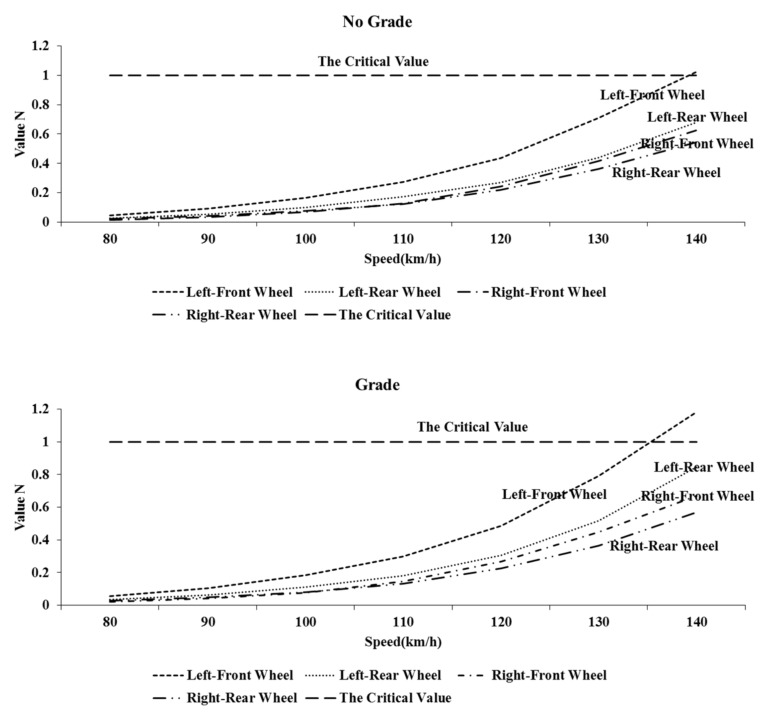
The critical skidding values of the SUV with or without grade.

As shown in Figure 6, the critical skidding condition of the SUV is achieved by increasing speed of the SUV with or without grade (case 4, in Table 4). The speed of the SUV was increased from 80 km/h, at intervals of 10 km/h, until a certain wheel was about to slide, which is at *N*≥1. It can be seen from Figure 6 that the value N of the left-front wheel (front-inner wheel) is the largest regardless of grade. When the vehicle speed was 140 km/h, the N values of the left-front wheel (front-inner wheel) reached critical condition. That is, when the vehicle was skidding, whether there was a grade or not, the left-front wheel (front inner wheel) was subject to skid first. The conclusion is consistent with that of Mavromatis obtained through calculating horsepower utilization factor [32]. In addition, it was found that in the curve with the grade, the speed of vehicle reaching critical skidding was lower, that is, the vehicle was easier to skid. Therefore, the left-front wheel (front-inner wheel) of the SUV was defined as the most critical wheel (wheel with priority to skid) when turning. The following studies were focused on the left-front wheel (front-inner wheel) of the SUV. The front wheel shows importance due to the front wheel drive configuration of the SUV. The internal wheel is due to the higher friction of the inner wheel caused by the lateral load transfer of the SUV negotiating on the curve. Although the skidding is not certain to occur when the condition of skidding is reached, it means that the vehicle is transiting to unstable lateral motion.

### 4.2. The Influence of Road Geometry on Vehicle Rollover and Skidding

Based on the “Highway Engineering Technology Standard” (JTG B 01-2014), the road segments of the most unfavorable geometric road combination with a design speed of 80 km/h are established, which are shown in Table 5 (Case 1–6) to study the effects of the super-elevation on skidding and rollover of a SUV. The radius of the circular curve was set to the minimum radius 250 m in the standard. The grade is at the minimum a value of 3% in the standard, and at the composite longitudinal slope (described in Formula 1) *I* ≤ 10.5%, thus, the maximum value of the super-elevation was 0.1. The road camber was set to the common value of 2%. Similarly, the unfavorable roads combination of horizontal curve, grade and super-elevation with a design speed of 80 km/h were established to study the effects of the grade on skidding and rollover of SUVs and are shown in in Table 5 (Case 7–11). The radius of circular curve is set to the minimum radius of 250 m required in the standard. In good friction pavement, the maximum grade is 6% required in the standard, and the super-elevation is the maximum value calculated according to I ≤ 10.5%.

**Figure 7:**
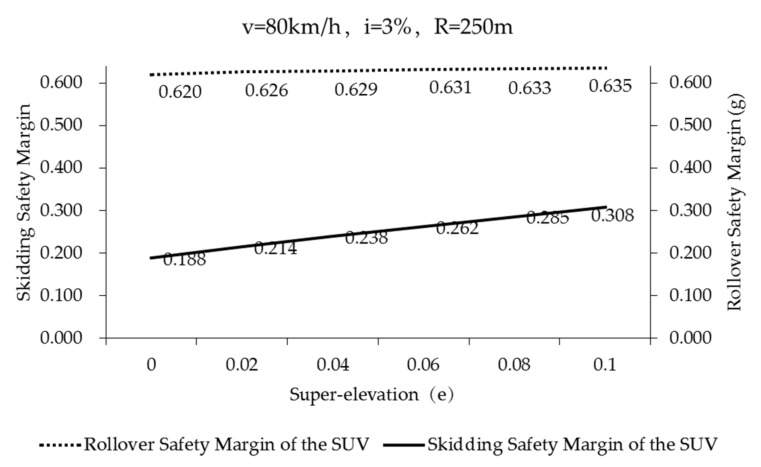
Friction demand of the SUV on good friction pavements.

The longitudinal and lateral friction values of the front-inner wheel of the SUV negotiating on good friction pavements (case 1–6 in Table 5) are shown in Figure 7. It can be seen that the lateral friction demand (dotted line) of the front-inner wheel is linearly related to the super-elevation approximately, which decreases with the increase of the super-elevation. The longitudinal frictional demand (Solid line) is positively correlated with super-elevation. Nevertheless, even at two extremes (e = 0, e = 0.1), the longitudinal frictional demand is 0.056 and 0.066, respectively. The difference is very small, thus, the correlation between longitudinal frictional demand and super-elevation is minor and can be neglected.

In the standard [33], the minimum radius formula of the point mass model during the curve negotiation is converted to the lateral friction demand formula as follows:

(17) $$f_{R}=\frac{V^{2}}{127R}-e$$

where *f_R_* is the lateral friction demand of the point mass model, *V (km/h)* is the vehicle design speed, *R (m)* is the radius of curves, and *e* is the super-elevation.

**Figure 8:**
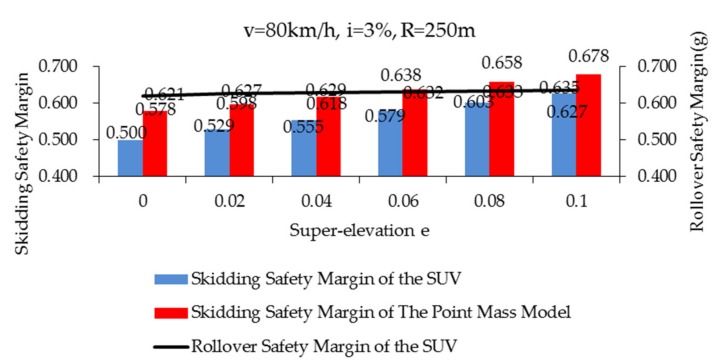
Skidding and safety rollover margin of vehicles on good friction pavement (*f_Tmax_* = 0.85) (Case 1–6, in Table 5).

The skidding safety margins of the point mass model during curve negotiation (case 1–6, in Table 5) (*f_Tmax_* = 0.85) are shown in Figure 8 and marked with a red strip. The blue strip represents the skidding safety margins (case 1–6, in Table 5) (*f_Tmax_* = 0.85) of the SUV. It was found that the skidding safety margins of the two models rises with the increase of super-elevation. Within the specified super-elevation range, the larger the super-elevation, the less likely the vehicle is to skid. The skidding safety margin of the point mass model is much larger than that of the SUV simulated by CarSim. The point mass model used in the standard does not take into account the lateral load transfer caused by vehicle roll and the influence of grade, thus, the vehicle’s skidding safety margin is relatively large. However, the skidding safety margin of the SUV is still very high even when the super-elevation is 0, which is 0.500. This means that on good friction pavement, the skidding risk of SUVs is very low while SUVs interact with the most unfavorable road geometry combination curve in the standard.

The black solid line in Figure 8 represents the safety margin of rollover of the SUV during curve negotiation (Case 1–6 in Table 5) (*f_Tmax_* = 0.85). It can be found that the super-elevation has little effect on the rollover safety margin of the SUV, so that it can be neglected. According to the literature [45], the lateral friction demand is a part of the lateral acceleration of the vehicle that will not be offset by the super-elevation. The scales of principal ordinate and secondary ordinate are the same in Figure 8. Through comparing the skidding safety margin (blue strip, principal ordinate) with the rollover safety margin (black solid line, secondary ordinate) of the SUV, it can be seen that both are very large. Therefore, if the SUV is passing through roads (*f_Tmax_* = 0.85) of the most unfavorable road geometry combination at the speed specified in the standard, both skidding and rollover will not occur easily (especially rollover). When comparing the safety margin of rollover and safety margin of skidding by CarSim model in Figure 8, the safety margin of rollover is always higher than that of skidding. Comparing the rollover safety margin and skidding safety margin by the point mass model; when the super-elevation is at *e* < 0.06, the safety margin of rollover is always higher than that of skidding. When the super-elevation is at *e ≥* 0.06, the rollover safety margin is slightly smaller than the skidding safety margin.

**Figure 9:**
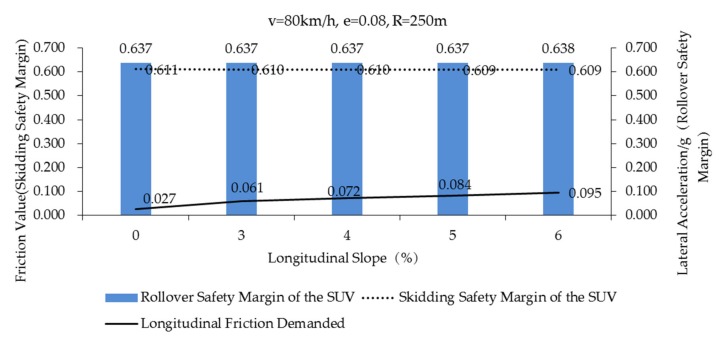
Skidding safety margin of the SUV, rollover safety margin of the SUV, longitudinal friction demanded of the SUV on good friction pavement (*f_Tmax_* = 0.85) (Case 7–11, in Table 5).

The rollover and skidding safety margin of the SUV during curve negotiation (case 7–11 in Table 5) (*f_Tmax_* = 0.85) is shown in Figure 9. It can be seen that the longitudinal friction demanded (black solid line) and grade are positive correlation. The safety margin of rollover (blue strip) is as much as the skidding safety margin (dotted line). It can be found that when the vehicle is negotiating a curve with good friction pavement, the grade has little effect on rollover and skidding of SUVs.

**Figure 10:**
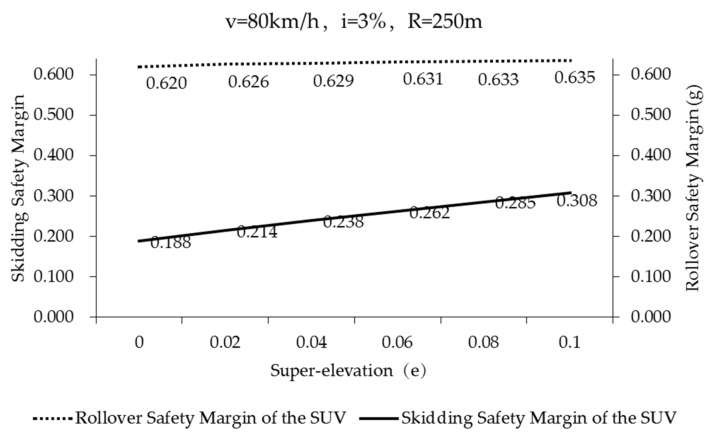
Skidding and rollover safety margin of the SUV on poor friction pavement (*f_Tmax_* = 0.50) (Case 1–6, in Table 5).

Figure 10 illustrates the safety margin of skidding and rollover of the SUV negotiating on poor friction asphalt pavement (Case 1-6, in Table 5) (*f_Tmax_* = 0.50). The solid line indicates the safety margin of skidding of the SUV. The dotted line indicates the safety margin of rollover of the SUV. The safety margin of skidding increases with the increase of super-elevation. When e=0, the safety margin of skidding is the smallest (*M_3_* = 0.188). In other words, the SUV can withstand the maximum additional lateral acceleration of 0.188g. When *e* = 0.1, the super-elevation balances partial lateral acceleration, and the skidding safety margin is at its largest *(M_3_* = 0.308). In other words, the SUV can withstand an additional lateral acceleration of up to 0.308g. The safety margin of rollover is less affected by super-elevation. Compared to the safety margin of rollover in Figure 8 (black solid line), the safety margin of rollover of SUVs on poor friction pavement in Figure 10 is slightly smaller. However, the safety margin of rollover is still very high, far greater than the safety margin of skidding in Figure 10.

From the above analysis, it can be found that rollover is not easy to occur on good and poor friction pavement for the SUV, even though the most unfavorable road geometry combination curve is designed. However, on poor friction pavement, the skidding safety margin of SUVs is significantly reduced with a minimum value of 0.188. In addition, in this paper the vehicle is steady turning on the established road, but when the vehicle runs on the curve, it is prone to over-steering or under-steering, which makes the skidding safety margin lower. That is, SUVs on poor friction pavement are prone to skidding under the most unfavorable geometric road combination curve.

### 4.3. Implementation of the Study

Factors such as the driver, vehicle type, center of mass, and wheels are considered in the closed-loop human-vehicle-road simulation model built in this paper. Compared with the mass point model used in the technical specification, the closed-loop human-vehicle-road model has a lower skidding safety margin. It was found that the front-inner wheel is the most unstable wheel in curve driving for the front-wheel driving vehicles. In future research on skidding, the front-inner wheel should be paid more attention to. When the vehicle drives strictly at the designed speed of the road, the longitudinal slope of the road has little influence on the skidding and rollover of the vehicle. When the vehicle is speeding, it is easier for the vehicle to reach the critical skidding state on the road with the longitudinal slope. Super-elevation has an effect on vehicle skidding and rollover. The larger the super-elevation is, the less likely the vehicle is to skid and rollover. The influence of super-elevation on skidding is greater than on rolling over. For the road of the most unfavorable road geometry combination in the standard, road alignment cannot cause SUVs with front-wheel drive to rollover. However, when the friction coefficient is also relatively low (*f_tmax_ = 0.50*), the skidding safety margin of vehicles can be relatively low and skidding can easily occur.

### 4.4. Policy Recommendations

The closed-loop human-vehicle-road model should replace in future technical specification of geometric road design, the traditional mass model so that a more comprehensive analysis (e.g., the driver, vehicle type, center of mass, and wheels) can be conducted for safety verification on the outcome of the geometric design.

Statistical analysis on accident data of multiple road sections needs to be conducted to better determine the appropriate threshold of vehicle safety to improve the safety and reliability of geometric road design.

According to the new model and safety threshold, old roads should be checked and assessed for its operations safety.

Road segments of the most unfavorable road geometry combination should be reconstructed or improved for safety consideration. New roads under design should be verified and assessed from a safety perspective. Any design of road segment that involves the most unfavorable combination of road geometry should be eliminated and redesigned before construction. If such road segments must be designed due to terrain or economic constraints, the vehicle speed limit or other operational treatments (e.g., traffic signs, color and/or high-friction pavement surface, etc.) must be strictly enforced.

## 5. Conclusions

In this paper, a closed-loop human-vehicle-road model is established by the vehicle dynamic simulation software, CarSim. At a design speed of 80 km/h, road segments of the most unfavorable road geometry combination are constructed for pavement surface with good friction (*f_Tmax_* = 0.85) and poor friction (*f_Tmax_* = 0.50). The study focuses on the influence of road geometry on rollover and skidding for a D-class mid-sized, front wheel drive SUV.

Studying the skidding safety margin of four wheels on road segments of the single most unfavorable geometry reveals the front-inner wheel of the SUV as the most critical wheel in skidding, whether a pavement grade exists or not.

The influence of grade on the safety margin of skidding is studied. It is discovered that the influence of grade on the safety margin of skidding is very little and can be negligible. The study of the influence of curve on the safety margin of skidding shows that the curve reduces greatly the safety margin of skidding.

Comparing the safety margin of skidding of the SUV and the point mass model on pavement surface with good friction (*f_Tmax_* = 0.85) reveals that safety margin of the point mass model is greater than that of the closed-loop human-vehicle-road model. Both models are affected by super-elevation; with the increase of super-elevation, the safety margin of skidding increases.

A comparison of the skidding safety margin with the rollover safety margin of the SUV reveals that the rollover safety margin is larger than that of the skidding safety margin (*f_Tmax_* = 0.80 and *f_Tmax_* = 0.50). The safety margin of rollover is always higher than that of skidding. That is, SUVs are more prone to skidding than to rollover. However, there is a bigger difference between the safety margin of skidding and rollover on pavement with poor friction (*f_Tmax_* = 0.50).

The minimum value of safety margin of skidding of the SUV is only 0.188 on the road of the most unfavorable road geometry combination (*f_Tmax_* = 0.50). That is, the SUVs are prone to skidding. This suggests that road segments of the most unfavorable road geometry combination should be avoided in a road’s geometric design. If such an unfavorable combination has to be included for some road segments in road geometric design due to terrain or economic constraints, then vehicle speed limit must be strictly controlled, especially for pavement with poor friction.

## Figures and Tables

**Table 1:** Simulation Parameters of SUV used in this study.

Parameter (unit)	Value
Sprung mass *m_s_* (*kg*)	1430
Full vehicle *m* (*kg*)	1610
Moment of inertia of sprung mass around X-axis *I_xx_* (*kg.m^2^*)	700.7
Moment of inertia of sprung mass around Y-axis *I_yy_* (*kg.m^2^*)	2059.2
Moment of inertia of sprung mass around Z-axis *I_zz_* (*kg.m^2^*)	2059.2
Horizontal distance between center of mass and front wheels *a* (*mm*)	1050
Horizontal distance between center of mass and rear wheels *b* (*mm*)	1610
Centroid height *h* (*mm*)	650
The height between the center of mass and the center of roll *h_c_* (*mm*)	573
Front wheelbase *c_f_* (*mm*)	1565
Rear wheelbase *c_r_* (*mm*)	1565
Distance from the tilt centers to the ground *h_f_* (*mm*) (front)	77
Distance from the tilt centers to the ground *h_r_* (*mm*) (rear)	130
Roll stiffness of suspensions *K_f_* (*N.m.rad^-1^*)(front)	181000
Roll stiffness of suspensions *K_r_* (*N.m.rad^-1^*)(rear)	57000
Damping coefficient of suspensions *b_f_* (*N.m.rad^-1^*)(front)	8430
Damping coefficient of suspensions b_r_ (*N.m.rad^-1^*)(rear)	8430
Wheel radius *R* (*mm*)	347
Wheel moment of inertia *I_w_* (*kg.m^2^*)	0.9

Note: SUV: D-class mid-sized sport utility vehicles with front-wheel drive.

**Table 2:** Fitting parameters of the ‘Magic formula’.

*a_0_*	*a_1_*	*a_2_*	*a_3_*	*a_4_*	*a_5_*	*a_6_*	*a_7_*	*a_8_*
1.30	−21.3	1101	1078	1.82	0.208	0	−0.354	0.707
*b_0_*	*b_1_*	*b_2_*	*b_3_*	*b_4_*	*b_5_*	*b_6_*	*b_7_*	*b_8_*
1.65	−22.1	1144	49.6	226	0.069	−0.006	0.056	0.486

**Table 3:** Good friction and poor friction values.

Good Friction Pavement(dry)		Poor Friction Pavement (Wet)	
*f_Tmax_*	*f_Rmax_*	*f_Tmax_*	*f_Rmax_*
0.85	0.78	0.5	0.46
*f_Rmax_ = 0.925f_Tmax_*			

**Table 4:** Road segments of Single Most Unfavorable Road Geometry.

Road Model	*R_min_(m)*(80 km/h)	Grade(Upslope)	Super-Elevation	Road Camber
Case 1	∞	0	0	0.02
Case 2	∞	6%	0	0.02
Case 3	250	0	0.1	0.02
Case 4	250	6%	0.08	0.02
*R_min_*: Radius of the Circular Curve				

**Table 5:** Road segments of the Most Unfavorable Road Geometry Combination Curve.

Road Models	*R_min_*(m)	Peak Friction Coefficient *f_Tmax_*	Grade (Upgrade)	Super-Elevation	Road Camber
Case 1	250	0.85 (good friction pavement);0.50 (poor friction pavement)	3%	0	0.02
Case 2	250		3%	0.02	0.02
Case 3	250		3%	0.04	0.02
Case 4	250		3%	0.06	0.02
Case 5	250		3%	0.08	0.02
Case 6	250		3%	0.1	0.02
Case 7	250	0.85 (good friction pavement)	0	0.08	0.02
Case 8	250		3%	0.08	0.02
Case 9	250		4%	0.08	0.02
Case 10	250		5%	0.08	0.02
Case 11	250		6%	0.08	0.02
f_Tmax_: Peak Friction Coefficient R_min_: Radius of Circular Curve					
